# Anti diabetic property of aqueous extract of *Stevia rebaudiana* Bertoni leaves in Streptozotocin-induced diabetes in albino rats

**DOI:** 10.1186/s12906-018-2245-2

**Published:** 2018-06-11

**Authors:** Uswa Ahmad, Rabia Shabir Ahmad

**Affiliations:** 1 Department of Food Science, Nutrition & Home Economics, Government College University, Allama Iqbal Road, Faisalabad, 38000 Pakistan; 2 Institute of Home and Food Sciences, Government College University, Faisalabad, 38000 Pakistan

**Keywords:** Diabetes, Fasting blood glucose, Insulin, HbA1c, Liver glycogen, Random blood glucose, *Stevia rebaudiana* bertoni, Stevioside

## Abstract

**Background:**

Stevia (*Stevia rebaudiana*) natural, non-caloric sugar substitute is rich source of pharmacologically important glycoside stevioside that is linked to the pathology and complications of diabetes.

**Methods:**

The current research was carried out to explore the anti-diabetic effect of aqueous extract of *Stevia rebaudiana* leaves in albino rats. For this purpose, diabetes was induced by administration of streptozotocin (40 mg/kg body weight, intraperitoneally). The diabetic rats were administered with aqueous stevia extract at different dose levels (200, 300, 400 and 500 ppm/kg b.w) for 8 weeks; the control rats were fed basal diet during this period.

**Results:**

Stevia aqueous extract improved caloric management and weight control by decreasing the feed intake and body weight gain. Furthermore, intake of stevia extract resulted in significant (*P* < 0.05) decrease in the random blood glucose level (− 73.24%) and fasting blood glucose (− 66.09%) and glycosylated (HbA1c) hemoglobin (5.32%) while insulin (17.82 μIU/mL) and liver glycogen (45.02 mg/g) levels significantly improved in the diabetic rats, compared with the diabetic and non-diabetic control rats after 8 weeks study period.

**Conclusions:**

It is concluded that aqueous extact of stevia has anti-diabetic effects in albino rats, and therefore could be promising nutraceutical therapy for the management of diabetes and its associated complications.

## Background

Diabetes mellitus is a group of metabolic diseases characterized by chronic hyperglycemia resulting from defects in insulin secretion, insulin action, or both [1]. According to World Health Organization Diabetes mellitus will become the seventh leading cause of death worldwide in 2030 [2]. Through proper diet, exercise and pharmacologic interventions, the incidence of diabetes can be overcome [3]. The pharmacological drugs used for the treatment of diabetes, are either too expensive or have certain adverse side effects. Therefore, for the treatment of diabetes mellitus many traditional plants have been preferred as natural source of drugs [4] because they are considered to be safe, less toxic than synthetic ones [5] and have strong antioxidant activities due to which these plants become more effective against diabetes [6]. *Stevia rebaudiana* Bertoni as a traditional plant is famous due to its sweet taste and beneficial effects in blood glucose regulation. *Stevia rebaudiana* Bertoni (family Asteraceae) popularly known as stevia, sweet weed, honey leaf and sweet herb of Paraguay [7]. Stevia leaves contained complex mixture of diterpene glycosides including stevioside, steviolbioside, rebaudiosides (A, B, C, D, E) and dulcoside A but the major sweet constituents are stevioside and rebaudioside A [8, 9]. Natural non-caloric sweetener stevioside (a major component of stevia) is 100–300 times sweeter than sucrose and have been extensively used as a non-caloric sugar substitute in many kinds of foods, medicine, beverage, cosmetics, wine making, household chemical industry and other food industries [10]. It possesses anti-hyperglycaemic, anti-hypertensive, anti-oxidant, anti-tumor, anti-diarrheal, diuretic, gastro and renal-protective and immunomodulatory properties [11]. The anti-hyperglycemic effect of *S. rebaudiana* was investigated in both rats and humans by [12, 13]. They mentioned that stevioside demonstrates a positive effect on hyperglycemia through decreasing the absorption of glucose in duodenum, glycogenolysis and gluconeogenesis.

As the synthetic drugs used for the treatment of diabetes result in many complications. Hence the use of natural source (*Stevia rebaudiana* Bertoni) for the treatment of diabetes is safe and non-carcinogenic [8, 9]. Hence, the present experiment was undertaken to study the antidiabetic effect of *S. rebaudiana* in albino rats.

## Methods

### Plant material

Stevia (*Stevia rebaudiana* Bertoni) leaves were collected from Ayub Agricultural Research Institute (AARI), Faisalabad (Reference no. 606/8). Stevia leaves were washed to remove the dirt, dust and foreign material adhered to the surface. After washing, leaves of stevia were air-dried under shade at room temperature and finely powdered with the help of grinder (MJ-176-NR-3899) [14].

### Stevia aqueous extract preparation

Stevioside was extracted from the dried ground leaves of stevia plant by using water extraction. The dried ground leaves of stevia were mixed with hot water (65 °C) at the ratio of 1:45 (*w*/*v*) [15]. The mixture was shaken properly and kept at room temperature for 24 h. It was stirred 2–3 times a day. After 24 h, mixture was filtered through What man filter paper and the filtrate was evaporated using rotary vacuum evaporator (EYELA N-1110S 115V) at 40–45 °C [14].

### Experimental animals

Sixty adult male albino rats of average weight 152.53 g were purchased from National Institute of Health, Islamabad, Pakistan, after getting permission from Institution of Animal Ethics Committee (IAEC). The rats were kept in stainless steel wire bottom cages under standard conditions (temperature 25 ± 2 °C and 60 ± 5% relative humidity with 12 h light-dark cycle) in environmentally controlled animal house of College of Pharmacology, Faculty of Science and Technology, Government College University, Faisalabad Pakistan. The rats were fed on the freshly prepared basal diet containing 65% starch, 10% casein, 10% corn oil, 4% salt mixture, 1% vitamins mixture and 10% cellulose [16] and distilled water for two week that meets their requirements for growing ad libitum.

### Induction of diabetes

The diabetes was induced in the rats by a single intraperitonial injection of STZ (40 mg/kg of body weight) freshly prepared in citrate buffer (0.1 M, pH 4.5), into the femoral vein of rats after an overnight fasting [17]. STZ-injected animals were given 20% glucose solution for 24 h to prevent initial drug-induced hypoglycemic mortality [5]. The normal control rats received only distilled water and standard diet.

Development of diabetes mellitus in the rats was confirmed by testing fasting blood glucose (FBG), after 72 h of STZ injection. The rats with FBG higher than 200 mg/dL were considered diabetic and were selected for the study [18].

### Animal groups and experimental design

Sixty male albino rats were divided into six groups of ten animals each. 1st and 2nd groups included normal (non-diabetic) and diabetic control rats respectively that received only distilled water that was free from impurities like dissolved salts and colloidal particles that can affect the results of the present research and standard diet throughout the whole trial. Diabetic rats consumed *Stevia rebaudiana* Bertoni aqueous extract dissolved at the levels of 200, 300, 400 and 500 ppm/kg b.w of albino rats in distilled water and administered orally as a daily dose for 8 weeks were included in 3rd, 4th, 5th and 6th groups respectively as shown in Table 1.

**Table 1:** Addition of aqueous Stevia extract in the distilled water of rats at different substitution levels

Non-diabetic rats	Diabetic rats				
N_0_	D_0_	D_1_	D_2_	D_3_	D_4_
Control (Basal diet+ distilled water)	Control (Basal diet+ distilled water)	Basal diet + 200 ppm SAE	Basal diet+ 300 ppm SAE	Basal diet+ 400 ppm SAE	Basal diet+ 500 ppm SAE

N_0_ = Basal diet and distilled water

D_0_ = Basal diet and distilled water

D_1_ = Basal diet and distilled water with 200 ppm Stevia leaf Aqueous extract

D_2_ = Basal diet and distilled water with 300 ppm Stevia leaf Aqueous extract

D_3_ = Basal diet and distilled water with 400 ppm Stevia leaf Aqueous extract

D_4_ = Basal diet and distilled water with 500 ppm Stevia leaf Aqueous extract

### Feed and water intake

Net feed intake of individual rat was calculated on daily basis by excluding left-over and collected spilled diet during the entire period to determine the effect of individual experimental diet. Water was provided with the help of graduated drinking bottles and its consumption was also measured on daily basis.

### Gain in body weight

Gain in body weight of individual rat in each group was estimated on weekly basis throughout the experimental period to find out the effect of individual diet on body weight using electronic weighing balance (KERN 440-35 N).

### Collection of serum of rats

For the serum, overnight fasted albino rats were killed using 0.4 mL of urethane anesthesia (25%) /100 g of body weight. Then blood was collected by cardiac puncture. After that serum was separated by centrifugation in the centrifuge machine (LABCENT 5000) at 3000 rpm for 15 min after allowing the blood to stand for at least 30 min at room temperature as per standard protocols [19].

### Analysis of serum biochemical profile of rats

Following analysis were made from the collected serum samples.

### Random blood glucose and fasting blood glucose levels

Fasting as well as random levels of glucose were estimated within 3 hours of sample collection by “GOD PAP Enzymatic Colorimetric Test Method” [20] on Humalyzer, 3000 (“Semi-automatic chemistry analyzer by Human, Germany, Model no. 16700”) by the use of standard kits. Effect of stevia aqueous extract on fasting blood glucose level as well as random blood glucose levels were observed at 1st, 2nd^,^ 3rd, 4th, 5th, 6th, 7th and 8th week of drug treatment in order to observe the variation in fasting and random blood glucose levels. For it blood was be taken by making a small cut at terminal tail vein of rats.

### Glycosylated hemoglobin (HbA1c) level

HbA1c in the blood was estimated by the method Nayak and Pattabiraman [21]. First lysed 5.5 mL of water with saline washed erythrocytes (0.5 mL), mixed and incubated for 15 min at 37 °C. The supernatant was discarded after the centrifugation of contents, then for the further process for estimation of HbA1c, 0.5 mL of saline was added and mixed. The contents were heated for 4 h at 100 °C after the addition of 0.02 mL of aliquot and 4 mL of oxalate hydrochloric solution. The solution was cooled and precipitated with 2 mL of 40% TCA. 0.5 mL of supernatant, 0.05 mL of 80% phenol and 3.0 mL of concentrated H_2_SO_4_ were added, after the centrifugation of the mixture. After 30 min, the color was developed that was read at 480 nm.

### Insulin level

The plasma insulin was assayed by Enzyme Linked Immunosorbent Assay (ELISA) method using Boehringer-Mannheim kit [22]. 0.1 mL of plasma was injected into the plastic tubes coated with monoclonal anti-insulin antibodies. To form anti-insulin antibody–POD conjugate, phosphate buffer and anti-insulin POD conjugate was added. Indicators reaction was formed by the addition of substrate chromogen solution. Then in the similar manner, a set of standards were also treated. The absorbance was read after the development of color at 420 nm.

### Liver glycogen

Liver glycogen level was measured according to the standard protocol [23]. Liver of both diabetic and non-diabetic rats was removed immediately at the end of the experiment and washed using ice-cold saline solution. Then hepatic tissues were minced and homogenized in hot ethanol (80%) at a tissue concentration of 100 mg/mL and centrifuged in the centrifuge machine (LABCENT 5000) at 9500 rpm for 20 min. 5 mL water and 6 mL of 52% perchloric acid were added. From it the residue was collected, dried and extracted. The collected material was centrifuged at 9500 rpm for 15 min for the recovery of supernatant. In the graduated test tube, 0.2 mL of supernatant, 1 mL distilled water and anthrone reagent (4 mL) was added, heated, cooled at room temperature and at 630 nm the intensity of the green to dark green color of the solution was recorded. From a standard curve prepared with standard glucose solution, glycogen content of the sample was determined.

### Statistical analysis

Results are expressed as mean ± standard deviation (SD). Analysis of variance (ANOVA) and least significance difference (LSD) were carried out on the result data at 95% confidence level using SPSS statistical software package, version 17 (SPSS Inc., Chicago).

## Results

**Fig. 1:**
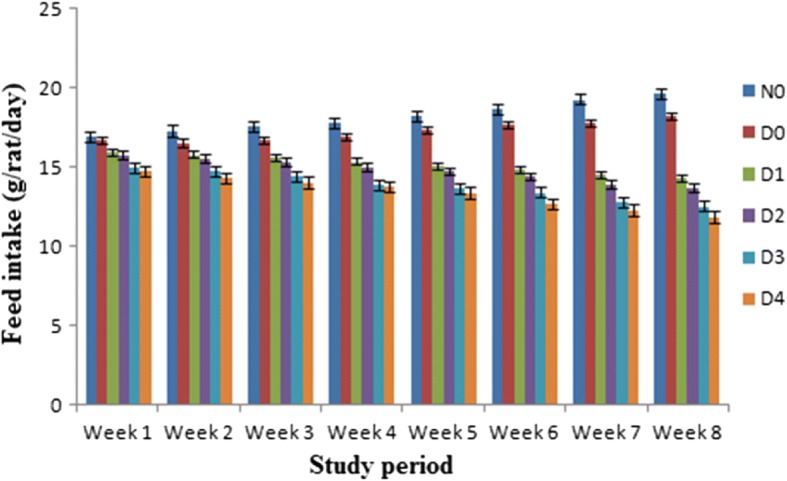
Feed intake (g) in normal and diabetic rats during 8 weeks (rat/week). Results are expressed as amount of feed intake levels of diabetic and non-diabetic rats (mean ± standard deviation (SD). *n* = 10). The feed intake of diabetic rats (D_1_, D_2_, D_3_ and D_4_) received stevia aqueous extract in different concentrations (200, 300, 400 and 500 ppm) respectively significantly (*P* < 0.05) decreased from non-diabetic (N_0_) and diabetic (D_0_) control groups

**Fig. 2:**
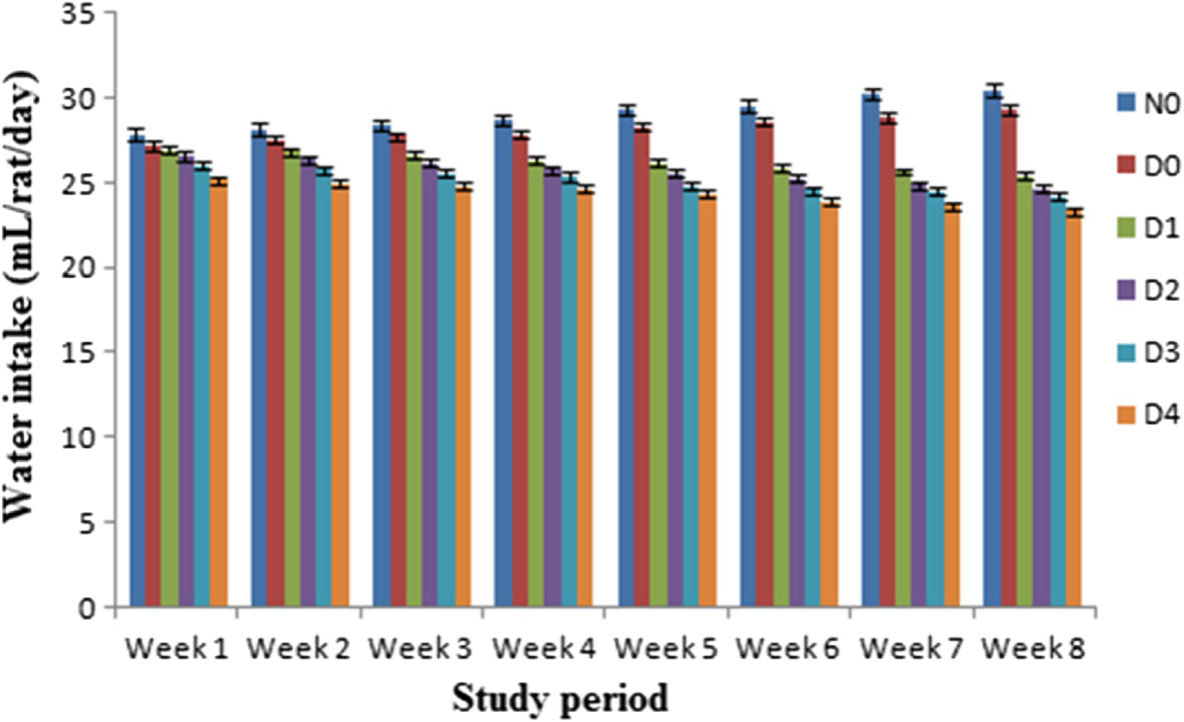
Water intake (mL) in normal and diabetic rats during 8 weeks (rat/week). Results are expressed as amount of water intake levels of diabetic and non-diabetic rats (mean ± standard deviation (SD). n = 10). The water intake of diabetic rats (D_1_, D_2_, D_3_ and D_4_) received stevia aqueous extract in different concentrations (200, 300, 400 and 500 ppm) respectively significantly (P < 0.05) decreased from non-diabetic (N_0_) and diabetic (D_0_) control groups

Means values for feed and water intakes in different groups of rats (per rat/day) have been shown graphically in Figs. 1 and 2. The results demonstrated that administration of stevia sweetener reduced the feed and water intakes in diabetic rats than N_0_ and D_0_. The highest feed and water intakes 14.57 g/rat/day, 29.82 mL/day and 13.14 g/rat/day, 28.95 mL/day respectively were observed in N_0_ (non-diabetic control) and D_0_ (diabetic control). While stevia sweetener at dose of 500 ppm/kg b.w showed the lowest amounts of feed and water intake (13.38 ± 0.98 g/rat/day) and (24.38 ± 0.58 mL/day) followed by 200 ppm/kg b.w (15.20 ± 1.00 g/day), (26.26 ± 0.53 mL/day), 300 ppm/kg b.w (14.81 ± 0.97 g/rat/day), (25.68 ± 0.60 mL/day) and 400 ppm/kg b. wt (13.82 ± 0.99 g/rat/day), (25.10 ± 0.64 mL/day) of stevia extracts respectively (Figs. 1 & 2).

Effect of administration of stevioside on the body weight of rats has been shown in Table 2. It is apparent from the results that the highest body weights (154.60 ± 4.02–185.90 ± 5.87 g/rat) and (153.22 ± 4.22–179.32 ± 4.55 g/rat) were observed in N_0_ (normal control) and D_0_ (diabetic control). While the lowest body weight was recorded in D_4_ (diabetic rats received 500 ppm/kg b.w aqueous stevia extract) (148.60 ± 7.02–120.81 ± 7.80 g/rat) as followed by D_1_ (152.12 ± 5.01–132.78 ± 4.32 g/rat), D_2_ (150.30 ± 6.33–128.70 ± 4.54 g/rat) and D_3_ (149.82 ± 6.88–124.32 ± 6.10 g/rat) during study period from 1st week to 8 weeks.

**Table 2:** Effect of *Stevia* aqueous extract on body weight of diabetic and non-diabetic rats

Diet groups	Week 0	Week 1	Weeks 2	Weeks 3	Weeks 4	Weeks 5	Weeks 6	Weeks 7	Weeks 8	Body weight gain (%)
N_0_	150.08 ± 9.02Ai	154.60 ± 4.02 Ah	158.15 ± 6.75Ag	161.18 ± 9.04Af	165.62 ± 9.22Ae	170.44 ± 9.33Ad	175.25 ± 9.21Ac	180.49 ± 5.43Ab	185.90 ± 5.87Aa	23.27
D_0_	150.06 ± 8.22Ai	153.22 ± 4.22 Ah	157.41 ± 5.43Ag	160.1 ± 9.32Af	164.00 ± 8.76Ae	167.30 ± 9.09ABd	171.54 ± 7.99Bc	175.82 ± 4.32Bb	179.32 ± 4.55Ba	19.48
D_1_	154.04 ± 9.44Ba	152.12 ± 5.01ABb	150.33 ± 6.22Bc	146.4 ± 8.02 Bd	143.21 ± 8.21Be	140.22 ± 9.21Bf	137.68 ± 8.76Cg	134.22 ± 4.87Ch	132.73 ± 4.32 Ci	−13.84
D_2_	153.02 ± 7.04Ba	150.30 ± 6.33Bb	147.32 ± 4.32BCc	143.2 ± 8.42Cd	140.00 ± 7.66Ce	137.40 ± 7.65Cf	134.68 ± 9.20Dg	131.60 ± 3.44CDh	128.70 ± 4.54CDi	−15.89
D_3_	153.05 ± 9.32Ba	149.82 ± 6.88Bb	146.56 ± 4.87BCc	143.9 ± 5.01Cd	140.90 ± 5.02Ce	136.32 ± 6.54Cf	133.95 ± 8.90Dg	129.53 ± 3.78Dh	124.32 ± 6.10Di	−18.75
D_4_	152.07 ± 9.11Ca	148.60 ± 7.02Bb	144.21 ± 3.21Cc	140.6 ± 5.11Dd	136.42 ± 6.05De	133.56 ± 5.65Df	130.21 ± 5.65Eg	125.87 ± 4.65Eh	120.81 ± 7.80Ei	−20.55

Values are mean ± standard deviation (SD) (n = 10)

Mean followed by different upper case letters (A, B, C, D) in the same columns represent significant difference (*P* < 0.05) treatment wise

Mean followed by different lower case letters (a, b, c, d) in the same rows represent significant difference (*P* < 0.05) among study periods (8 weeks)

N_0_ = Non-diabetic rats given basal diet and distilled water

D_0_ = Diabetic rats given basal diet and distilled water

D_1_ = Diabetic rats given basal diet and distilled water with 200 ppm Stevia leaf aqueous extract

D_2_ = Diabetic rats given basal diet and distilled water with 300 ppm Stevia leaf aqueous extract

D_3_ = Diabetic rats given basal diet and distilled water with 400 ppm Stevia leaf aqueous extract

D_4_ = Diabetic rats given basal diet and distilled water with 500 ppm Stevia leaf aqueous extract

Regarding the body weight gain %, highest percentage (23.27%) and (19.48%) was observed in N_0_ and D_0_. When diabetic rats were given stevia sweetener then their body weight gain % decreased by − 13.84, − 15.89, − 18.75 and − 20.55% respectively after 8 weeks (Table 2).

Table 3 represents the random blood glucose levels of normal and diabetic rats, affected by different levels of stevia aqueous extracts. From the results, it was observed that random blood glucose (RBG) levels of N_0_ and D_0_ increased from (82.73 ± 2.91 to 89.25 ± 2.76 mg/dL) and (342.1 ± 1.22 to 391.22 ± 1.65 mg/dL) respectively at the beginning of the study to end of trail respectively. However, the RBG levels of D_1_, D_2_, D_3_ and D_4_ decreased from 332.23 ± 1.34, 330.23 ± 1.44, 327.88 ± 1.23 and 326.44 ± 1.65 mg/dL at 1st week to 94.43 ± 1.23, 93.29 ± 1.54, 91.22 ± 1.87 and 90.77 ± 1.27 mg/dL respectively at 8th week (Table 3). Stevia extract decreased the random blood glucose % levels of groups D_1_, D_2_, D_3_ and D_4_ by − 71.74, − 72.25, − 73.08 and − 73.24% respectively after 8 weeks (Table 3).

**Table 3:** Random blood glucose levels (mg/dL) of normal and diabetic rats

Diet groups	Week 0	Week 1	Weeks 2	Weeks 3	Weeks 4	Weeks 5	Weeks 6	Weeks 7	Weeks 8	RBG %
N_0_	80.72 ± 3.82Ec	82.73 ± 2.91Dc	83.4 ± 2.62Ebc	83.7 ± 2.44Fbc	84.9 ± 2.54Fb	85.4 ± 2.66Fb	87.3 ± 2.86Fa	88.4 ± 2.77Fa	89.25 ± 2.76Ca	7.88
D_0_	340.1 ± 2.32Dd	342.1 ± 1.22Ad	348.7 ± 1.32Ad	354.3 ± 1.43Acd	360.9 ± 1.66Ac	366.2 ± 1.55Ac	273.3 ± 1.54Ac	381.2 ± 2.89Ab	391.22 ± 1.65Aa	15.04
D_1_	334.23 ± 1.99Ca	332.23 ± 1.34Bb	320.22 ± 1.44Bc	278.88 ± 1.65 Bd	256.66 ± 1.67Be	166.67 ± 1.43Bf	143.33 ± 1.87Bg	122.23 ± 1.78Bh	94.43 ± 1.23Bi	−71.74
D_2_	336.23 ± 2.94Ba	330.23 ± 1.44BCb	312.22 ± 1.43Cc	262.22 ± 1.34Cd	240.44 ± 1.88Ce	153.32 ± 1.77Cf	130.98 ± 1.65Cg	118.87 ± 1.54Ch	93.29 ± 1.54Bi	−72.25
D_3_	338.88 ± 3.77ABa	327.88 ± 1.23Cb	310.88 ± 1.65CDc	251.42 ± 1.23Dd	232.22 ± 1.98De	140.98 ± 1.87Df	122.21 ± 1.87Dg	112.32 ± 1.56Dh	91.22 ± 1.87BCi	−73.08
D_4_	339.22 ± 4.32Aa	326.44 ± 1.65Cb	308.65 ± 1.23Cc	243.32 ± 1.77Ed	221.32 ± 1.80Ee	127.76 ± 1.45Ef	116.54 ± 1.98Eg	107.65 ± 1.32Eh	90.77 ± 1.27 Ci	−73.24

Values are mean ± standard deviation (SD) (n = 10)

Mean followed by different upper case letters (A, B, C, D) in the same columns represent significant difference (*P* < 0.05) treatment wise

Mean followed by different lower case letters (a, b, c, d) in the same rows represent significant difference (*P* < 0.05) among study periods (8 weeks)

As presented in Table 4, there was significant (*P* < 0.05) increase in the fasting blood glucose level of the diabetic control group rats, relative to the normal control group. However, this was significantly (P < 0.05) restored toward normal in the diabetic rats given stevia aqueous extract, as indicated by the decrease in their fasting blood glucose levels from the 1st week to the 8th week. According to results highest level of fasting blood glucose (306.4 ± 2.65 mg/dL) was recorded in D_0_ (diabetic control group rats). While fasting blood glucose levels of diabetic rats received stevia aqueous extract significantly decreased from 90.70 ± 2.98 (D_1_) to 88.22 ± 2.97 (D_4_) mg/dL. The fasting blood glucose % levels of groups D_1_, D_2_, D_3_ and D_4_ decreased by − 64.87, − 65.28, − 65.96 and − 66.09% respectively after 8 weeks (Table 4).

**Table 4:** Fasting blood glucose levels (mg/dL) of normal and diabetic rats

Diet groups	Week 0	Week 1	Weeks 2	Weeks 3	Weeks 4	Weeks 5	Weeks 6	Weeks 7	Weeks 8	FBG %
N_0_	80.20 ± 3.47Dc	80.23 ± 2.94Dc	81.22 ± 2.92Fc	82.32 ± 2.89Fbc	82.30 ± 2.86Fbc	83.44 ± 2.65Fb	84.55 ± 2.90Fb	86.65 ± 2.78Fa	87.77 ± 2.99Ca	9.39
D_0_	262.3 ± 2.34Ai	266.4 ± 2.54 Ah	269.8 ± 2.87Ag	275.6 ± 2.67Af	280.8 ± 1.22Ae	286.6 ± 1.45Ad	292.3 ± 1.45Ac	298.6 ± 1.32Ab	306.4 ± 2.65Aa	16.81
D_1_	258.22 ± 1.99Ca	255.32 ± 2.94Ba	245.45 ± 2.34Bb	221.73 ± 2.93Bc	212.99 ± 2.98 Bd	165.55 ± 1.34Be	132.22 ± 1.65Bf	112.55 ± 1.87Bg	90.70 ± 2.98Bh	−64.87
D_2_	258.18 ± 2.94Ca	253.22 ± 2.91BCa	237.77 ± 2.97Cb	215.55 ± 2.76Cc	180.99 ± 2.94Cd	155.54 ± 2.87Ce	122.87 ± 2.91Cf	106.66 ± 1.87Cg	89.64 ± 2.81Bh	−65.28
D_3_	259.88 ± 3.77Ba	242.21 ± 2.42Cb	220.88 ± 2.90Dc	212.34 ± 2.21Dd	160.66 ± 2.93De	134.44 ± 2.93Df	115.54 ± 1.22Dg	104.44 ± 1.89Dh	89.44 ± 2.88Bi	−65.96
D_4_	260.02 ± 4.32Ba	240.22 ± 2.76Cb	215.55 ± 2.91Ec	180.88 ± 2.76Ed	145.55 ± 2.65Ee	122.32 ± 2.32Ef	108.78 ± 1.32Eg	102.22 ± 1.34Eh	88.22 ± 2.97BCi	−66.09

Values are mean ± standard deviation (SD) (n = 10)

Mean followed by different upper case letters (A, B, C, D) in the same columns represent significant difference (*P* < 0.05) treatment wise

Mean followed by different lower case letters (a, b, c, d) in the same rows represent significant difference (*P* < 0.05) among study periods (8 weeks)

**Fig. 3:**
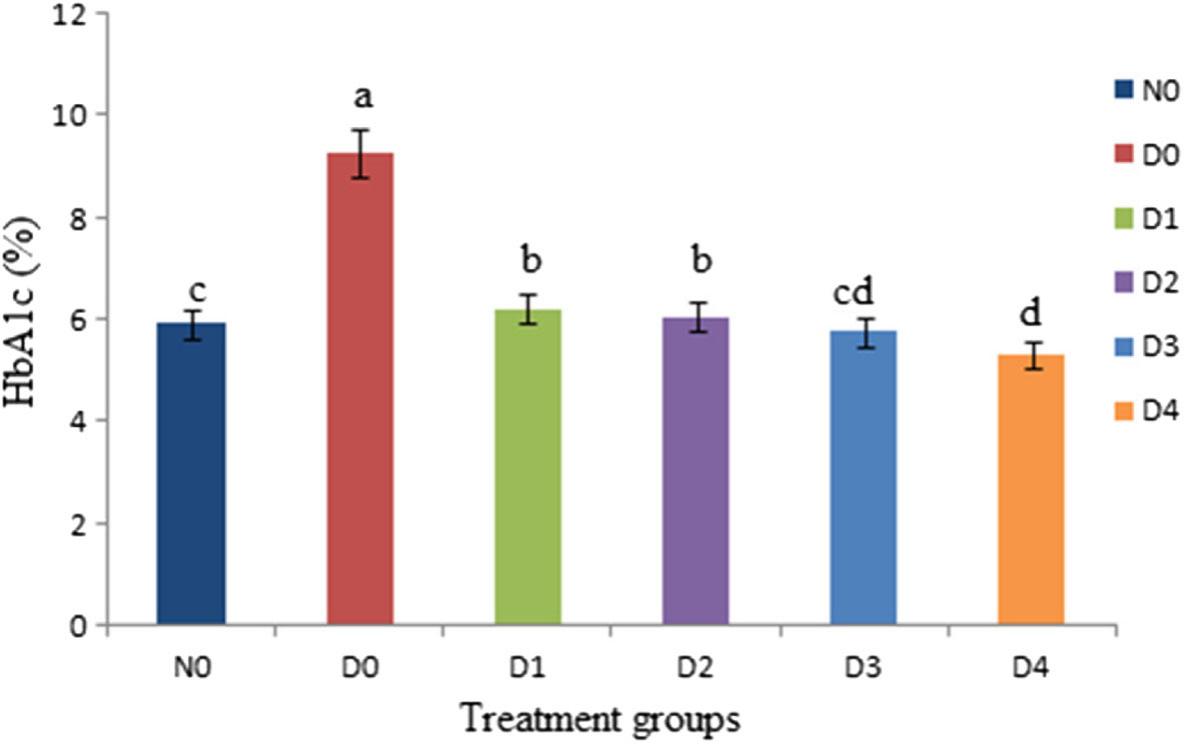
Effect of Stevia aqueous extract on the glycosylated hemoglobin (HbA1c) level of the rats. Results are expressed as percentage of HbA1c levels of diabetic and non-diabetic rats (mean ± standard deviation (SD). n = 10). a, b, c, d represent significant difference (P < 0.05) in HbA1c levels treatment wise. HbA1c levels of diabetic rats (D_1_, D_2_, D_3_ and D_4_) received stevia aqueous extract in different concentrations (200, 300, 400 and 500 ppm) respectively significantly (*P* < 0.05) decreased as compared diabetic (D_0_) control groups and near to N_0_

The glycosylated hemoglobin (HbA1c) level of the rats is shown in Fig. 3. According to results HbA1c level (9.27 ± 1.09%) of D_0_ significantly (*P* < 0.05) increased than N_0_ (5.92 ± 1.02%). But as compared to the D_0_, diabetic groups D_1_, D_2_, D_3_ and D_4_ received stevia aqueous extract had significantly (*P* < 0.05) lower HbA1c levels (6.22 ± 1.11%, 6.06 ± 1.08%, 5.77 ± 1.06% and 5.32 ± 1.00%) respectively; indicating that the stevia extract decrease the glycosylation of hemoglobin.

**Fig. 4:**
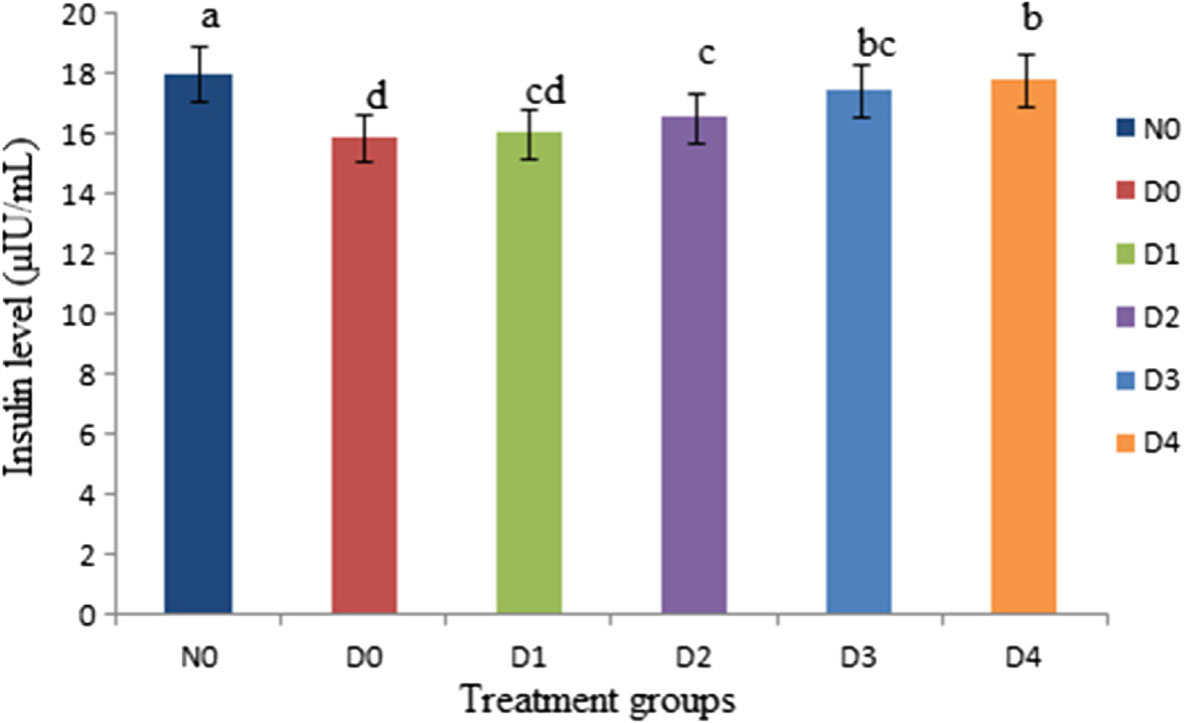
Effect of Stevia aqueous extract on insulin levels of different groups of rats. Results are expressed as concentration of insulin levels of diabetic and non-diabetic rats (mean ± standard deviation (SD). *n* = 10). a, b, c, d represent significant difference (*P* < 0.05) in insulin levels treatment wise. The insulin levels of diabetic rats (D_1_, D_2_, D_3_ and D_4_) received stevia aqueous extract in different concentrations (200, 300, 400 and 500 ppm) respectively significantly (*P* < 0.05) increased as compared diabetic (D_0_) control groups and near to N_0_

The insulin levels of diabetic and normal rats are shown in Fig. 4. According to results the insulin levels of diabetic D_0_ (15.89 ± 1.22 μIU/mL) control group decreased as compared to N_0_ (18.02 ± 1.44 μIU/mL). The results of this study concluded that diabetic rats given stevioside mixed in distilled water increased the levels of serum insulin. The results further demonstrated that given stevia aqueous extracts at different dose levels improved significantly (*P* < 0.05) from 16.04 ± 1.24 to 17.82 ± 1.33 μIU/mL (D_1_ to D_4_) (Fig. 4).

**Fig. 5:**
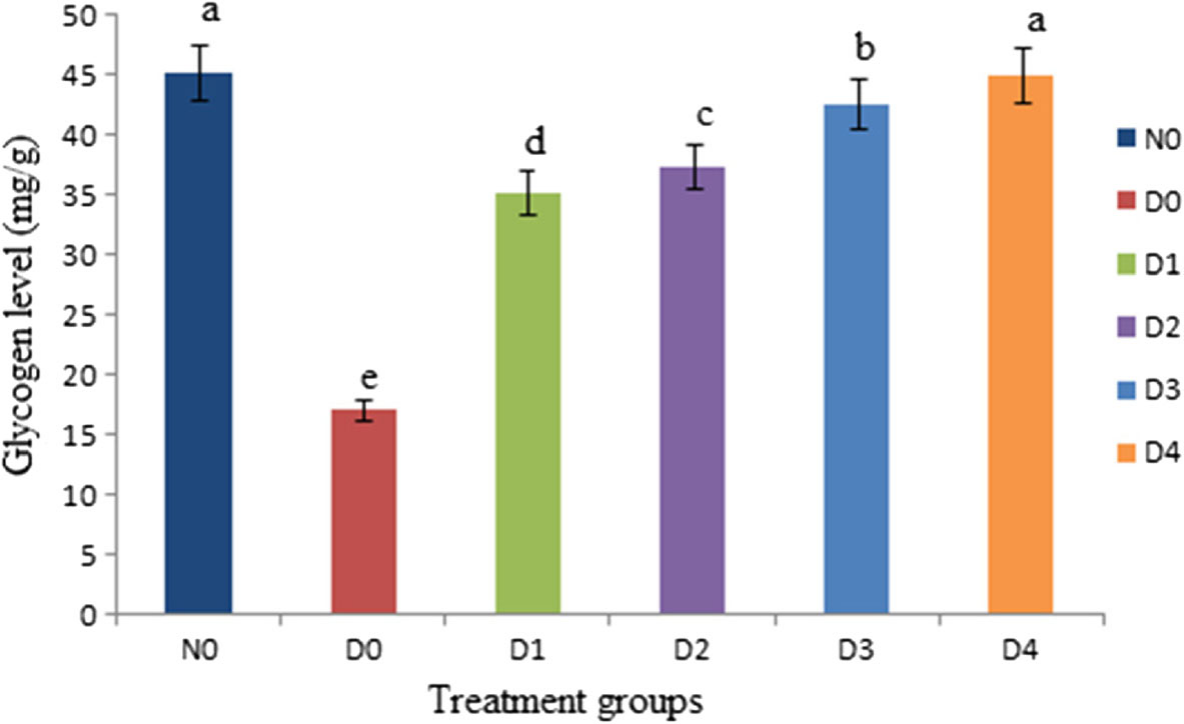
Effect of Stevia aqueous extract on the glycogen level of the rats. Results are expressed as concentration of glycogen levels of diabetic and non-diabetic rats (mean ± standard deviation (SD). n = 10). a, b, c, d represent significant difference (P < 0.05) in insulin levels treatment wise. The glycogen levels of diabetic rats (D_1_, D_2_, D_3_ and D_4_) received stevia aqueous extract in different concentrations (200, 300, 400 and 500 ppm) respectively significantly (P < 0.05) increased as compared diabetic (D_0_) control groups and near to N_0_

The liver glycogen level of the rats is shown in Fig. 5. In this study, glycogen level of D_0_ (17.07 ± 1.35 mg/g) decreased significantly (P < 0.05) compared to the N_0_ (45.22 ± 2.22 mg/g) (Fig. 5). However, the diabetic rats received stevia aqueous extracts (200, 300, 400 and 500 ppm/kg) significantly (*P* < 0.05) increased the liver glycogen levels (35.27 ± 2.12, 37.43 ± 2.14, 42.66 ± 2.20 and 45.02 ± 2.24 mg/g) (Fig. 5).

## Discussion

In this study, we evaluated the anti-diabetic activity of aqueous extract of stevia in diabetic albino rats as previous researches confirmed its pharmacological importance due to presence of glycosides like stevioside in it. Administration of aqueous stevia extract orally at different concentrations (200, 300, 400 and 500 mg/kg) for 8 weeks, significantly decline the feed and water intakes of diabetic albino rats. Stevia a low-caloric sweetener may reduce the feed and water intake and not promote weight gain because they do not stimulate the appetite [24]. Similarly, stevia sweetener at doses of 25, 250, 500 and 1000 mg/kg b. w may also reduce the feed intake in adult female wistar strain rats [25].

The results indicated that aqueous extract from leaves of *Stevia rebaudiana*, produced a significant (*P* < 0.05) dose-dependent reduction in body weight (Table 2) and body weight gain percentage of the rats treated with Stevia extract (Table 3) as compared to N_0_. The highest gain in body weight was noticed in N_0_ while lowest was recorded in D_4_. Stevioside in the diet lowered the blood glucose level. The body weight of rats might reduce due to lower metabolisation of diet glucose or decrease amount of rat’s food consumption [26]. This reduction in weights of rats receiving stevia extract may be due to high amount of stevioside that reduced the food intake of rats [27]. This finding is collaborated with the previous researches which proved a positive association between the decrease of body weight gain percent and the decline in feed intake and dose of stevioside given to the rats [28–30].

This study depicted that different concentrations of stevia extract had a good efficacy in controlling diabetes with an excellent control of random and fasting blood glucose level in diabetic rats at study period of 8 weeks. Previous study showed that stevioside was able to regulate blood glucose levels by enhancing not only insulin secretion and sensitivity but also insulin utilization in insulin deficient rats which was due to decreased PEPCK gene expression in rat liver [31]. According to another study, stevia extract may contain some biomolecules that may sensitize the insulin receptor to insulin or stimulates the β-cells of islets of langerhans to release insulin which may finally lead to improvement of carbohydrate metabolizing enzymes towards the reestablishment of normal blood glucose level [32]. *Stevia rebaudiana* leaves extract decreased the random and fasting blood glucose levels of rats by revitalizing the β-cells of pancreas thus reactivated the glycogen synthase system by improving insulin secretion and liver glycogen level [25, 27, 33, 34]. These results are in agreement with Awney et al. [27]; Abo Elnaga et al. [25]; Assaei et al. [30] and Akbarzadeh et al. [34] who also observed that stevia aqueous extract lowered the random and fasting blood glucose levels in diabetic rats due to more insulin secretion and increased glycogen level.

The diabetic rats treated with stevia aqueous extract exhibited HbA1c values near normal levels (≥6.5% (48 mmol/mol) as a result of improved glycemic control due to initiation of glycogen production framework of the extract. The decrease of HbA1c showed that the ability of extract to control the diabetes [33].

These results are in accordance with Prasad et al. [35] and Rao et al. [36] who demonstrated the anti-diabetic effects of ethanolic extract of the roots of *Chonemorpha fragrans* and combination of herbal product (*Curcuma longa* and *Eugenia jambolana*) in streptozotocin- induced diabetic rats and concluded that both have a good efficacy in controlling diabetes.

The serum insulin level in the diabetic control group decreased due to STZ that resulted in diabetes by the rapid depletion of β-cells, which reduced the insulin release. An insufficient release of insulin causes hyperglycemia, which results in oxidative damage by the generation of reactive oxygen species and the development of diabetic complications [37]. When stevia aqueous extracts at different dose levels were given to the diabetic albino rats then their insulin levels improved significantly (Fig. 4) due to the presence of natural components (stevioside) in stevia leaves that are related to inhibition of hepatic expression of phosphoenolpyruvate carboxykinase and gluconeogenesis coupled with stimulation of hepatic glycogen synthesis that resulted in increase of insulin secretion and insulin sensitivity [38]. Evidence from other studies revealed that stevia aqueous extract elevate the insulin level due to stevioside that acts on pancreatic tissue, exerts beneficial anti-hyperglycemic effects through the PPARγ-dependent mechanism [34, 30].

The results are in collaborations with the studies conducted by Shivanna et al. [39]; Saleh et al. [40] and Abou khalil et al. [41] who concluded that aqueous extracts of *Stevia rebaudiana* leaves and Desert date (*Balanites aegyptiaca*) and Parsley (*Petroselinum sativum*) stevioside normalize the pancreatic cell function by restoring the insulin immune reactivity in STZ-induced diabetic rats.

The reduction in insulin release and liver glycogen level of diabetic control group rats are due to STZ (a known diabetogen**)** used for induction of diabetes in rats that brings about the destruction of β- cells of the islets of Langerhans [42]. However, the diabetic rats received stevia aqueous extracts (200, 300, 400 and 500 ppm/kg) were able to significantly (*P* < 0.05) improve the liver glycogen levels (Fig. 5). Stevioside (sweetener) present in stevia extract acts directly on pancreatic beta cells and resulted in increase of insulin secretion [12]. Increased level of Insulin enhances intracellular glycogen deposition by stimulating activities of glycogen synthase and inhibiting glycogen phosphorylase [38].

Similar results were observed by previous researchers who found that *Plectranthus esculenthus* extracts and *Mangifera indica* kernel flour-supplemented diet restored the liver glycogen levels in STZ induced diabetic rats [42, 43].

## Conclusions

The present study suggests that aqueous extract from stevia leaves may decrease the random blood glucose level and fasting blood glucose and glycosylated (HbA1c) hemoglobin while insulin and liver glycogen levels significantly increased of the diabetic rats, compared with the diabetic and non-diabetic control rats after 8 weeks study period. It is concluded that aqueous extact of stevia with concentration 500 ppm/kg body weight of rats showed best results of all the parameters determined. It is understood from the results that stevia extract has anti-diabetic effects in albino rats, and therefore could be used as natural anti-diabetic drug for the treatment of diabetes and its associated complications.

## Abbreviations

BWG %: Body weight gain percentage
FBG: Fasting blood glucose
HbA1c: Glycosylated hemoglobin
PEPCK: Phosphoenolpyruvate carboxykinase
RBG: Random blood glucose
STZ: Streptozotocin
